# Trends in the Prevalence of Overweight and Obesity among Chinese Preschool Children from 2006 to 2014

**DOI:** 10.1371/journal.pone.0134466

**Published:** 2015-08-12

**Authors:** Yanyu Xiao, Yijuan Qiao, Lei Pan, Jin Liu, Tao Zhang, Nan Li, Enqing Liu, Yue Wang, Hongyan Liu, Gongshu Liu, Guowei Huang, Gang Hu

**Affiliations:** 1 Department of Nutrition and Food Science, School of Public Health, Tianjin Medical University, Tianjin, China; 2 Tianjin Women’s and Children’s Health Center, Tianjin, China; 3 Chronic Disease Epidemiology Laboratory, Pennington Biomedical Research Center, Baton Rouge, LA, United States of America; Institute of Preventive Medicine, DENMARK

## Abstract

**Objective:**

To examine the trends in the prevalence of overweight and obesity among preschool children from 2006 to 2014.

**Methods:**

A total of 145,078 children aged 3–6 years from 46 kindergartens finished the annual health examination in Tianjin, China. Height, weight and other information were obtained using standardized methods. Z-scores for weight, height, and BMI were calculated based on the standards for the World Health Organization (WHO) child growth standards.

**Results:**

From 2006 to 2014, mean values of height z-scores significantly increased from 0.34 to 0.54, mean values of weight z-scores kept constant, and mean values of BMI z-scores significantly decreased from 0.40 to 0.23. Mean values of height z-scores, weight z-scores, and BMI z-scores slightly decreased among children from 3 to 4 years old, and then increased among children from 4 to 6 years old. Between 2006 and 2014, there were no significant changes in prevalence of overweight (BMI z-scores >2 SD) and obesity (BMI z-scores >3 SD) among 3–4 years children. However, prevalence of obesity (BMI z-scores >2 SD) increased from 8.8% in 2006 to 10.1% in 2010, and then kept stable until 2014 among 5–6 years children. Boys had higher prevalence of obesity than girls.

**Conclusions:**

Mean values of BMI z-scores decreased from 2006 to 2014 among Chinese children aged 3–6 years old due to the significant increase of height z-scores. Prevalence of obesity increased from 2006 to 2010, and then kept stable until 2014 among children aged 5–6 years. The prevalence of obesity was higher in boys than in girls.

## Introduction

In China, following rapid economic development from 1980s, Chinese people have rapidly changed their lifestyles tending towards a more sedentary and a high-energy/high-fat diet lifestyle [1]. This change has resulted in an increased prevalence of overweight and obesity in China [1]. Overweight and obesity increase the risks of coronary heart disease, hypertension, type 2 diabetes, dyslipidemia, several types of cancers, and premature mortality [2].

Obesity has become an important public problem not only in Chinese adults but also in children [3]. The prevalence of obesity among Chinese children in the coastal big cities has reached the average level of the developed countries[4]. A recent meta-analysis has shown that the prevalence of obesity among Chinese children and adolescents increased from 0.4% in 1981–1985 to 7.5% in 2006–2010 [5]. Another study from six Chinese National Surveys on Students Constitution and Health has reported that the prevalence of obesity in children aged 7–18 years increased rapidly from 0.2% in 1985 to 8.1% in 2010 [6]. It has been suggested that developing effective prevention and intervention programs for the children at formative pre-school years (3–6 years old) might be an important step in combating the childhood obesity epidemic because eating and physical activity habits that contribute to later obesity become established during these formative years and these habits are more malleable at this time than in later childhood [7, 8]. However, research in this age-group is limited, especially on long-term trend of prevalence of overweight and obesity. The aim of the present study was to examine the trends in the prevalence of overweight and obesity among pre-school children aged 3–6 years between 2006 and 2014 in Tianjin, China.

## Methods

### Study sample

**Fig 1:**
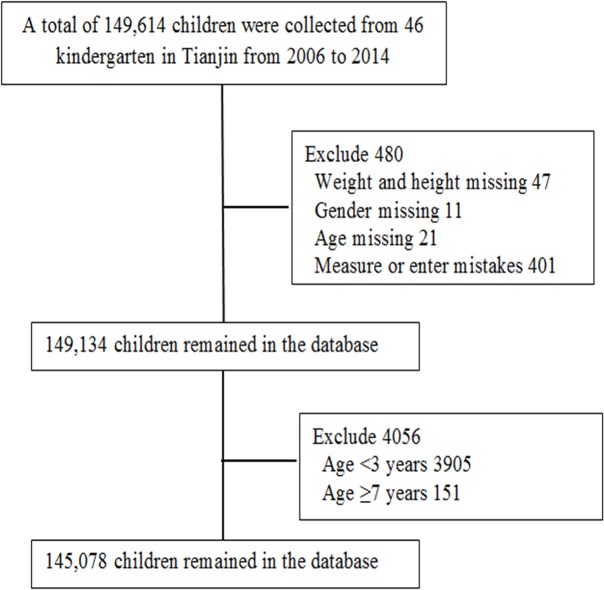
Detailed flow chart for data cleaning procedure.

Tianjin is the fourth largest city with over 14.1 million residents in northern China. Tianjin consists of 16 county-level administrative areas, including six central urban districts, one new urban district, six suburban districts and five rural districts. Most of children aged 3–6 years attend local kindergarten schools. Tianjin Women and Children’s Health Center is responsible for implementation and promotion of children’s health in the kindergarten school. In Tianjin, kindergarten schools generally include three grades (3–6 years old). Each grade has several classes, and each class has about 20 to 30 children. In order to monitor the growth and development of children in Tianjin, a stratified cluster sampling was employed to obtain a random sample of children in Tianjin. We selected 2–4 kindergarten schools from each urban and suburban district and 1 kindergarten school from each rural district in each year survey. From 2006 to 2014, 46 kindergarten schools were monitored continuously. All children in these kindergartens were invited to take the annual physical examination. The participation rates of the children in kindergarten schools varied from 97.1% to 98.9% in different study years. A total of 149,614 children finished the survey. After excluding 4536 children with incomplete data, more than 7 years old or less than 3 years old, the present analyses included 145,078 children (Fig 1). The dataset for the present analysis comes from the regular annual health examination. In the annual health examination, parents in more than 80% of kindergartens gave written informed consents, and parents in the rest of kindergartens gave verbal informed consents. If the parents agreed their children to take the annual health examination, the parents of the children paid the health examination fee to the kindergarten school. Thus we can record each participant’s consent from his/her parent’s health examination payment. The Tianjin Women’s and Children’s Health Center Institutional Review Board has agreed to use this written informed consent or verbal informed consent procedure from all participants involved in our study. The study and analysis plan were approved by the Tianjin Women and Children's Health Center Institutional Review Board.

### Measurements

The survey included questionnaire and anthropometric measurements. A questionnaire was given to kindergarten teachers and kindergarten teachers filled in at kindergartens. The questionnaire included questions on child's name, birth date, gender, and personal unique code. All these data were collected when the children entered the kindergarten schools at the beginning of the school year. Health care records for children from birth to 7 years old have been collected and available in electronic form since 2010 [9–11].

From March to May in each year, professionally trained staff measured height and weight using the standardized protocol. Height and weight were measured without shoes and in light clothing. Standing height was measured to the nearest 0.1 cm using a Stadiometer (SZG-180, Shanghai Zhengdahengqi Company, Shanghai, China). Weight was measured to the nearest 0.01 kg using a digital scale (TCS-60, Tianjin weighting apparatus, Tianjin, China). Body mass index (BMI) was calculated by dividing weight in kilograms by the square of height in meters. Z-scores for weight, height, and BMI were calculated based on the standards for the World Health Organization (WHO) child growth standards (2–5 years old), and WHO child growth reference (5–19 years old) [12, 13]. Overweight and obesity were defined based on the WHO’s different recommended cut-offs. Among 3–4 years old children, overweight was defined as a BMI z- score >2 SD, and obesity was defined as a BMI z-score >3 SD[14]. Among 5–6 years old children, overweight was defined as a BMI z-score >1 SD, and obesity was defined as a BMI z-score >2 SD [12].

### Statistical analyses

Differences in the mean values of height z-scores, weight z-scores, and BMI z-scores between sexes, and the linear trends in the mean values of height z-scores, weight z-scores, and BMI z-scores across different study years or different age groups were tested using analysis of variance. Differences in the prevalence of overweight and obesity between sexes and ages were tested by Chi-square tests. The linear trends in the prevalence of overweight and obesity across different study years were tested by the logistic regression. The criterion for statistical significance was <0.05. All statistical analyses were performed with SPSS for Windows, version 21.0 (Statistics 21, SPSS, IBM, USA) or SAS for Windows, version 9.4 (SAS Institute, Cary, NC).

## Results

A total of 145,078 annual health examination records were collected from 2006 to 2014, which included 76,389 boys (52.7%) and 68,689 girls (47.3%) (Table 1). The annual numbers of children were generally stable, from 14,257 in 2006 to 18,603 in 2014. The average age was 4.9±0.9 years. The proportion of boys declined slightly over years. There were significant differences in age distributions from 2006 to 2014.

**Table 1:** Sample sizes by sex and age in the children at 3–6 years in Tianjin from 2006 to 2014.

	Year									Total	P value
	2006	2007	2008	2009	2010	2011	2012	2013	2014		
No. of participants	14,257	13,804	13,843	15,245	15,726	17,100	17,811	18,689	18,603	145,078	
Boys (n, %)	7,624(53.5)	7,374(53.4)	7,324(52.9)	8,002(52.5)	8,224(52.3)	8,927(52.2)	9,349(52.5)	9,823(52.6)	9,742(52.4)	76,389(52.7)	0.012 ^a^
Mean values of age(Years)	4.8 (0.9)	5.0 (0.9)	4.9 (1.0)	4.8 (0.9)	4.8 (0.9)	4.9 (0.9)	4.9 (0.9)	4.9 (0.9)	4.9 (0.9)	4.9 (0.9)	0.002^a^
Age group (n, %)											<0.001^b^
3 years	3,125(21.9)	1,862(13.5)	3,173(22.9)	3,218(21.1)	3,151(20.0)	3,478(20.3)	3,232(18.1)	3,417(18.3)	3,301(17.7)	27,957(19.3)	
4 years	4,695(32.9)	5,242(38.0)	3,558(25.7)	6,014(39.4)	5,490(34.9)	5,595(32.7)	6,695(37.6)	6,337(33.9)	6,723(36.1)	50,349(34.7)	
5 years	4,544(31.9)	4,546(32.9)	5,080(36.7)	3,755(24.6)	5,430(34.5)	5,318(31.1)	5,372(30.2)	6,369(34.1)	5,775(31.0)	46,189(31.8)	
6 years	1,893(13.3)	2,154(15.6)	2,032(14.7)	2,258(14.8)	1,655(10.5)	2,709(15.8)	2,512(14.1)	2,566(13.7)	2,804(15.1)	20,583(14.2)	

Data are means (SD) or number (percentage)

^a^ P value for trend.

^b^ P value for difference.

During 2006–2014, mean values of height z-scores significantly increased among both boys (from 0.39 to 0.61) and girls (from 0.27 to 0.47), mean values of weight z-scores kept constant, and mean values of BMI z-scores significantly decreased among both boys (from 0.53 to 0.34) and girls (from 0.26 to 0.10) (Table 2). Boys had higher mean values of height z-scores, weight z-scores, and BMI z-scores than girls (Table 2). Mean values of height z-scores, weight z-scores, and BMI z-scores slightly decreased among children from 3 to 4 years old, and then increased among children from 4 to 6 years old (Table 2).

**Table 2:** Mean values of z-scores for weight, height and BMI in the children at 3–6 years in Tianjin from 2006 to 2014.

	Year									Total	P value for trend
	2006	2007	2008	2009	2010	2011	2012	2013	2014		
Height z-scores											
Total samples	0.34	0.36	0.41	0.43	0.47	0.50	0.48	0.53	0.54	0.46	<0.001
Age group (Years)											
3	0.30	0.32	0.35	0.39	0.43	0.43	0.40	0.46	0.45	0.40	<0.001
4	0.25	0.31	0.34	0.38	0.40	0.43	0.41	0.45	0.49	0.39	<0.001
5	0.41	0.38	0.48	0.47	0.55	0.55	0.58	0.58	0.60	0.52	<0.001
6	0.43	0.46	0.47	0.53	0.50	0.64	0.58	0.66	0.66	0.56	<0.001
P value for trend	<0.001	<0.001	<0.001	<0.001	<0.001	<0.001	<0.001	<0.001	<0.001	<0.001	
Sex											
Boys	0.39	0.42	0.49	0.51	0.54	0.57	0.56	0.60	0.61	0.53	<0.001
Girls	0.27	0.28	0.33	0.34	0.39	0.42	0.40	0.45	0.47	0.38	<0.001
P value	<0.001	<0.001	<0.001	<0.001	<0.001	<0.001	<0.001	<0.001	<0.001	<0.001	
Weight z-scores											
Total samples	0.48	0.50	0.53	0.52	0.53	0.54	0.49	0.53	0.49	0.51	0.317
Age group (Years)											
3	0.49	0.49	0.51	0.47	0.46	0.47	0.40	0.46	0.36	0.46	<0.001
4	0.40	0.45	0.44	0.48	0.46	0.46	0.41	0.41	0.42	0.44	0.158
5	0.52	0.51	0.57	0.54	0.62	0.58	0.59	0.59	0.54	0.57	0.002
6	0.61	0.58	0.64	0.65	0.64	0.72	0.63	0.72	0.72	0.66	<0.001
P value for trend	<0.001	<0.001	<0.001	<0.001	<0.001	<0.001	<0.001	<0.001	<0.001	<0.001	
Sex											
Boys	0.61	0.62	0.68	0.66	0.68	0.68	0.64	0.66	0.63	0.65	0.323
Girls	0.34	0.35	0.38	0.35	0.37	0.39	0.34	0.37	0.35	0.36	0.713
P value	<0.001	<0.001	<0.001	<0.001	<0.001	<0.001	<0.001	<0.001	<0.001	<0.001	
BMI z-scores											
Total samples	0.40	0.40	0.41	0.37	0.35	0.34	0.28	0.29	0.23	0.33	<0.001
Age group (Years)											
3	0.45	0.43	0.44	0.33	0.29	0.30	0.22	0.25	0.12	0.31	<0.001
4	0.37	0.40	0.35	0.37	0.32	0.30	0.25	0.21	0.18	0.30	<0.001
5	0.38	0.39	0.39	0.36	0.39	0.34	0.32	0.33	0.24	0.35	<0.001
6	0.49	0.42	0.49	0.45	0.45	0.46	0.38	0.43	0.44	0.44	0.084
P value for trend	0.639	0.690	0.167	0.001	<0.001	<0.001	<0.001	<0.001	<0.001	<0.001	
Sex											
Boys	0.53	0.52	0.54	0.51	0.50	0.47	0.41	0.42	0.34	0.46	<0.001
Girls	0.26	0.27	0.26	0.21	0.20	0.19	0.14	0.15	0.10	0.19	<0.001
P value	<0.001	<0.001	<0.001	<0.001	<0.001	<0.001	<0.001	<0.001	<0.001	<0.001	

According to the WHO’s recommended cut-offs for classifying overweight and obesity, the definitions of overweight and obesity are different between children under the age of 5 years and those over the age of 5 years. Therefore, we have presented the results into two different age groups (3–4 years old in Table 3 and 5–6 years old in Table 4). Between 2006 and 2014, there were no significant changes in prevalence of overweight (BMI z-scores >2 SD) and obesity (BMI z-scores >3 SD) among 3–4 years old children (Table 3). However, prevalence of obesity (BMI z-scores >2 SD) increased from 8.8% in 2006 to 10.1% in 2010, and then kept stable until 2014 among 5–6 years old children (Table 4). Boys had higher prevalence of overweight (3–4 years old: 7.4% vs. 3.9%; 5–6 years old: 29.6% vs.19.4%) and obesity (3–4 years: 2.5% vs. 1.0%; 5–6 years: 13.3% vs.5.7%) than girls.

**Table 3:** Prevalence of overweight and obesity in the children at 3–4 years old in Tianjin from 2006 to 2014.

	Year									Total	P value for trend
	2006	2007	2008	2009	2010	2011	2012	2013	2014		
**Overweight** ^a^											
Total samples	5.5	5.9	5.6	5.9	6.1	6.0	5.6	5.4	5.6	5.7	0.574
Age group (years)											
3	5.3	5.0	5.3	4.2	5.1	5.4	4.3	4.8	4.3	4.9	0.072
4	5.6	6.2	5.8	6.9	6.7	6.3	6.3	5.7	6.2	6.2	0.788
P value	0.638	0.079	0.321	<0.001	0.004	0.063	<0.001	0.070	<0.001	<0.001	
Sex											
Boys	6.8	7.5	7.7	8.2	7.9	7.3	7.1	6.8	7.0	7.4	0.220
Girls	3.9	4.0	3.3	3.5	4.2	4.5	4.1	3.8	4.0	3.9	0.308
P value	<0.001	<0.001	<0.001	<0.001	<0.001	<0.001	<0.001	<0.001	<0.001	<0.001	
**Obesity** ^b^											
Total samples	1.7	1.6	1.8	1.9	2.0	1.8	1.7	1.7	1.8	1.8	0.572
Age group (years)											
3	1.4	1.1	1.5	1.2	1.6	1.1	1.0	1.4	1.1	1.3	0.317
4	1.9	1.8	2.0	2.3	2.3	2.3	2.1	1.9	2.2	2.1	0.325
P value	0.117	0.034	0.092	<0.001	0.032	<0.001	<0.001	0.061	<0.001	<0.001	
Sex											
Boys	2.2	2.2	2.8	2.9	2.8	2.6	2.4	2.4	2.5	2.5	0.821
Girls	1.1	0.9	0.7	0.8	1.1	1.0	0.9	1.1	1.1	1.0	0.333
P value	<0.001	<0.001	<0.001	<0.001	<0.001	<0.001	<0.001	<0.001	<0.001	<0.001	

^a^ Overweight was defined as a BMI z- score >2 SD

^b^ Obesity was defined as a BMI z-score >3 SD

**Table 4:** Prevalence of overweight and obesity in the children at 5–6 years old in Tianjin from 2006 to 2014.

	Year									Total	P value for trend
	2006	2007	2008	2009	2010	2011	2012	2013	2014		
**Overweight** ^a^											
Total samples	24.2	24.1	25.0	24.6	25.6	25.1	24.5	25.6	24.3	24.8	0.294
Age group (years)											
5	23.1	23.6	23.9	23.2	25.2	23.5	23.7	24.5	22.3	23.7	0.867
6	26.7	25.3	27.7	27.0	27.0	28.2	26.0	28.2	28.4	27.2	0.049
P value	0.002	0.130	0.001	0.001	0.147	<0.001	0.026	<0.001	<0.001	<0.001	
Sex											
Boys	29.0	29.0	29.6	30.1	30.6	30.2	29.1	30.0	28.6	29.6	0.994
Girls	18.6	18.5	19.5	18.5	20.1	19.4	19.1	20.6	19.5	19.4	0.052
P value	<0.001	<0.001	<0.001	<0.001	<0.001	<0.001	<0.001	<0.001	<0.001	<0.001	
**Obesity** ^b^											
Total samples	8.8	8.6	9.1	9.4	10.1	10.1	10.2	10.7	10.1	9.7	<0.001
Age group (years)											
5	7.8	8.1	8.3	8.8	9.7	9.1	9.6	10.0	8.7	9.0	<0.001
6	11.4	9.7	11.3	10.6	11.4	12.0	11.4	12.4	12.8	11.5	0.001
P value	<0.001	0.028	<0.001	0.019	0.48	<0.001	0.014	0.001	<0.001	<0.001	
Sex											
Boys	12.4	12.0	12.4	13.1	14.0	13.7	13.3	14.6	13.6	13.3	<0.001
Girls	4.7	4.7	5.3	5.3	5.8	6.1	6.6	6.2	6.1	5.7	<0.001
P value	<0.001	<0.001	<0.001	<0.001	<0.001	<0.001	<0.001	<0.001	<0.001	<0.001	

^a^ Overweight was defined as a BMI z- score >1 SD

^b^ Obesity was defined as a BMI z-score >2 SD

During 2006–2014, the prevalence of obesity significantly increased in 5–6 years old children who lived in the new urban and suburban districts and did not change in central urban districts and rural areas (Table A in S1 File). The children who lived in the new urban had the highest prevalence of overweight and obesity Trends in the prevalence of overweight and obesity from 2006 to 2014 were also presented using the IOTF reference (Table B in S1 File)

## Discussion

This study indicated that there were no significant changes in prevalence of overweight and obesity among Chinese children aged 3–4 years old. However, the prevalence of obesity increased from 8.8% in 2006 to 10.1% in 2010, and then stayed stable until 2014 among children aged 5–6 years old. The prevalence of obesity was higher in boys than in girls.

The prevalence of obesity among preschool children varied in different reports [15–20]. A study from 450 national representative cross-sectional surveys from 144 counties reported that the prevalence of childhood overweight/obesity(BMI z-scores >2SD) among 0–5 years was 4.9% in Asia in 2010, which was lower than that in our study [21]. Another study from 2002 China Nutrition and Health Survey indicated that the prevalence of overweight/obesity (BMI z-scores >2SD) was 5.4% among 0–6 years old children, which was similar to our 3–4 years group but far lower than our 5–6 years groups [3]. A report from 2005 Chinese National Survey on Students Constitution and Health found that Tianjin had the highest prevalence of childhood obesity (11.97%) in 29 provinces [22].

Reports from the Chinese National Survey on Students Constitution and Health demonstrated that the prevalence of obesity (Chinese BMI criteria) among 7–12 years old children increased from 0.2% in 1985 to 6.8% in 2005 among boys and from 0.2% in 1985 to 3.7% in 2005 among girls [23]. One national epidemiological survey showed a fast increased trend in prevalence of child obesity from 1986 to 2006 among Chinese children under 7 years old [24]. It is well known that a sedentary lifestyle and unhealthy dietary habits contribute to obesity [25]. China was going through transitions from a traditional lifestyle to a westernized lifestyle in the past thirty years. With modernization and industrialization physical activities including occupational, commuting and leisure-time physical activities are reduced substantially [26]. At the same time, Chinese children have gradually changed their food habits from traditional high-carbohydrate diets to high-fat/high salt diets [27, 28]. The change towards a high-fat dietary pattern and an increase in sedentary habit may lead to an increased prevalence of overweight/obesity in Chinese children. However, our study found that there were no significant changes in prevalence of obesity from 2006 to 2014 among 3–4 years old children, and the prevalence of obesity increased from 2006 to 2010, and then kept stable until 2014 among 5–6 years children. This may be due to social, parents and school increasing awareness of childhood obesity and paying more attention to childhood obesity prevention and intervention in recent years. The significant decreases in BMI z-scores among pre-school children from 2006 to 2014 in the present study are due to the significant increase of children’s height z-scores. The results from the present study are the same as the findings of another report from seven large surveys conducted in China, which found a steady increase in children’s height between 1975 and 2010 [29].

Recent data have shown that excessive weight gain and/or overweight/obesity in the first several years of life are associated with increased risks of subsequent obesity and unfavorable cardiometabolic outcomes in childhood, adolescence, and adulthood [30–32]. The present study found that mean values of weight z-scores and BMI z-scores significantly increased among children from 4 to 6 years old. Some studies have shown that diet, physical activity, and behavioral modifications are effective in reducing childhood obesity [33, 34], however, obesity is difficult to reverse in older children and adults [8]. Thus childhood obesity experts suggest that prevention of childhood obesity should start at formative pre-school years (3–6 years old). In Tianjin, most of children at 3–6 years old stay at kindergarten schools. These children eat three meals and two snacks, and do regular exercise at kindergarten schools, which account for major daily energy intake and energy expenditure. Thus developing effective prevention and intervention programs for the children in the kindergarten might be an important step in combating the childhood obesity epidemic.

There are several strengths and limitations in our study. First, this study reported data on a large group of preschool children in 9 consecutive study years, from 2006 to 2014. Second, we had data on standardized measurement of children’s weight and height. There are two limitations in this study. First, this is a cross-sectional study. We could not longitudinally evaluate the trend of obesity among the same children in kindergartens. We will assess this question in the future research. Second, we did not collect the information of socioeconomic factors, parental body size, parents’ lifestyle habits, children’s nutrition and physical activity, etc. We could not determine the reasons for the changed trend in the prevalence of obesity among preschool children.

## Conclusions

In conclusion, the present study indicated that there were no significant changes in prevalence of overweight and obesity among Chinese children aged 3–4 years old, and prevalence of obesity increased from 8.8% in 2006 to 10.1% in 2010, and then stayed stable until 2014 among children aged 5–6 years old. The prevalence of obesity was higher among boys than among girls. The results of this study warrant that obesity prevention and intervention programs should focus on pre-school children.

## Supporting Information

S1 File Prevalence of overweight and obesity from 2006 to 2014 by different region in the children at 3–6 years in Tianjin(Table A).Prevalence of overweight and obesity using IOTF definition by different age, sex and region from 2006 to 2014 in the children at 3–6 years in Tianjin(Table B) (DOCX)

S2 File Tianjin preschool children physical examination database(2006–2014) (XLSX)
