# Supplementary material for: Trends in the Prevalence of Overweight and Obesity among Chinese Preschool Children from 2006 to 2014
Source: PLoS One. 2015 Aug 12;10(8):e0134466. doi: 10.1371/journal.pone.0134466 (PMC4534378; doi:10.1371/journal.pone.0134466)
Supplement: S1 File — (DOCX) [file pone.0134466.s001.docx]

Table A. Prevalence of overweight and obesity from 2006 to 2014 by different regions in the children at 3-6 years in Tianjin

|  | Year | | | | | | | | | Total | P value  for trend |
| --- | --- | --- | --- | --- | --- | --- | --- | --- | --- | --- | --- |
|  | 2006 | 2007 | 2008 | 2009 | 2010 | 2011 | 2012 | 2013 | 2014 |  |  |
| 3-4 years old |  |  |  |  |  |  |  |  |  |  |  |
| Overweight ^a^ |  |  |  |  |  |  |  |  |  |  |  |
| Central urban | 5.5 | 5.0 | 4.3 | 5.7 | 5.7 | 5.8 | 5.2 | 5.5 | 5.1 | 5.4 | 0.742 |
| New urban | 5.8 | 6.9 | 7.5 | 7.5 | 6.9 | 6.4 | 6.2 | 7.1 | 6.8 | 6.8 | 0.964 |
| Suburban | 5.1 | 6.3 | 5.5 | 4.8 | 6.1 | 6.0 | 5.6 | 4.1 | 5.1 | 5.4 | 0.164 |
| Rural area | 5.5 | 4.9 | 4.8 | 6.1 | 5.3 | 5.2 | 5.8 | 3.9 | 5.8 | 5.3 | 0.766 |
| P value | 0.811 | 0.032 | <0.001 | 0.001 | 0.237 | 0.652 | 0.445 | <0.001 | 0.024 | <0.001 |  |
| Obesity ^b^ |  |  |  |  |  |  |  |  |  |  |  |
| Central urban | 1.5 | 1.1 | 1.2 | 1.6 | 1.5 | 1.6 | 1.5 | 1.5 | 1.8 | 1.5 | 0.129 |
| New urban | 1.8 | 2.2 | 2.4 | 2.3 | 2.4 | 1.9 | 2.1 | 2.4 | 2.1 | 2.2 | 0.752 |
| Suburban | 1.7 | 1.8 | 1.9 | 1.9 | 2.3 | 2.0 | 1.4 | 1.4 | 1.6 | 1.8 | 0.221 |
| Rural area | 1.9 | 1.0 | 1.1 | 2.3 | 2.0 | 1.6 | 2.2 | 1.8 | 2.0 | 1.8 | 0.381 |
| P value | 0.807 | 0.017 | 0.018 | 0.269 | 0.065 | 0.745 | 0.102 | 0.022 | 0.476 | <0.001 |  |
| 5-6 years old |  |  |  |  |  |  |  |  |  |  |  |
| Overweight ^c^ |  |  |  |  |  |  |  |  |  |  |  |
| Central urban | 25.1 | 27.6 | 25.0 | 25.4 | 25.8 | 25.2 | 24.5 | 26.6 | 23.0 | 25.3 | 0.059 |
| New urban | 26.5 | 24.0 | 28.1 | 24.7 | 28.2 | 27.0 | 26.7 | 27.2 | 26.9 | 26.7 | 0.229 |
| Suburban | 21.5 | 21.3 | 22.6 | 24.3 | 24.3 | 24.2 | 23.1 | 24.7 | 24.4 | 23.5 | 0.002 |
| Rural area | 22.1 | 20.2 | 23.3 | 22.8 | 21.2 | 21.7 | 22.2 | 20.8 | 21.9 | 21.7 | 0.874 |
| P value | 0.002 | <0.001 | 0.001 | 0.603 | 0.003 | 0.028 | 0.018 | 0.001 | 0.003 | <0.001 |  |
| Obesity ^d^ |  |  |  |  |  |  |  |  |  |  |  |
| Central urban | 8.9 | 9.5 | 9.4 | 9.0 | 9.6 | 9.6 | 10.2 | 10.3 | 9.4 | 9.6 | 0.150 |
| New urban | 9.5 | 8.3 | 10.6 | 10.6 | 11.8 | 12.0 | 10.5 | 11.1 | 11.2 | 10.7 | 0.004 |
| Suburban | 8.0 | 7.8 | 7.9 | 9.4 | 9.5 | 9.3 | 10.3 | 10.8 | 10.3 | 9.3 | <0.001 |
| Rural area | 9.1 | 8.2 | 7.8 | 8.2 | 9.0 | 8.6 | 8.7 | 10.5 | 8.7 | 8.9 | 0.335 |
| P value | 0.463 | 0.202 | 0.021 | 0.251 | 0.037 | 0.005 | 0.550 | 0.771 | 0.072 | <0.001 |  |

^a^ Overweight was defined as a BMI z- score >2 SD

^b^ Obesity was defined as a BMI z-score >3 SD

^c^ Overweight was defined as a BMI z- score >1 SD

^d^ Obesity was defined as a BMI z-score >2 SD

Table B. Prevalence of overweight and obesity using IOTF definition by different age, sex and region from 2006 to 2014 in the children at 3-6 years in Tianjin

|  | Year | | | | | | | | | Total | P value  for trend |
| --- | --- | --- | --- | --- | --- | --- | --- | --- | --- | --- | --- |
|  | 2006 | 2007 | 2008 | 2009 | 2010 | 2011 | 2012 | 2013 | 2014 |  |  |
| Overweight |  |  |  |  |  |  |  |  |  |  |  |
| Total | 14.4 | 14.7 | 15.0 | 14.3 | 14.8 | 14.9 | 14.3 | 14.8 | 13.9 | 14.6 | 0.183 |
| Age group (years) |  |  |  |  |  |  |  |  |  |  |  |
| 3 | 11.2 | 10.4 | 11.1 | 9.4 | 10.1 | 10.4 | 8.2 | 9.0 | 7.8 | 9.7 | <0.001 |
| 4 | 12.2 | 13.2 | 12.6 | 13.8 | 12.5 | 12.2 | 12.3 | 11.7 | 11.2 | 12.4 | 0.001 |
| 5 | 16.2 | 16.3 | 16.9 | 16.2 | 18.0 | 16.9 | 17.2 | 18.1 | 16.5 | 17.0 | 0.064 |
| 6 | 20.9 | 18.8 | 20.8 | 19.8 | 21.3 | 22.3 | 20.8 | 22.2 | 22.3 | 21.1 | 0.004 |
| P value | <0.001 | <0.001 | <0.001 | <0.001 | <0.001 | <0.001 | <0.001 | <0.001 | <0.001 | <0.001 |  |
| Sex |  |  |  |  |  |  |  |  |  |  |  |
| Boys | 15.7 | 15.8 | 16.5 | 16.0 | 16.3 | 15.9 | 15.4 | 16.0 | 15.1 | 15.8 | 0.183 |
| Girls | 13.0 | 13.5 | 13.3 | 12.5 | 13.3 | 13.8 | 13.0 | 13.5 | 12.6 | 13.2 | 0.722 |
| P value | <0.001 | <0.001 | <0.001 | <0.001 | <0.001 | <0.001 | <0.001 | <0.001 | <0.001 | <0.001 |  |
| Region |  |  |  |  |  |  |  |  |  |  |  |
| Central urban | 15.3 | 15.0 | 14.2 | 14.5 | 14.4 | 14.3 | 13.6 | 14.6 | 12.2 | 14.2 | <0.001 |
| New urban | 15.4 | 15.7 | 17.9 | 16.2 | 16.5 | 16.3 | 15.6 | 16.6 | 16.5 | 16.3 | 0.575 |
| Suburban | 12.5 | 14.3 | 14.0 | 13.1 | 14.3 | 14.7 | 14.0 | 14.2 | 14.0 | 13.9 | 0.085 |
| Rural area | 14.4 | 12.3 | 12.9 | 12.4 | 13.0 | 13.6 | 13.5 | 12.7 | 13.5 | 13.2 | 0.963 |
| P Value | <0.001 | 0.026 | <0.001 | <0.001 | 0.002 | 0.018 | 0.016 | <0.001 | <0.001 | <0.001 |  |
| Obesity |  |  |  |  |  |  |  |  |  |  |  |
| Total | 4.2 | 4.3 | 4.4 | 4.4 | 5.2 | 5.0 | 5.0 | 5.0 | 4.8 | 4.7 | <0.001 |
| Age group (years) |  |  |  |  |  |  |  |  |  |  |  |
| 3 | 2.3 | 2.1 | 2.2 | 2.0 | 2.4 | 2.0 | 2.2 | 2.2 | 2.0 | 2.2 | 0.554 |
| 4 | 3.7 | 3.5 | 3.7 | 3.9 | 4.3 | 4.2 | 4.1 | 3.6 | 3.9 | 3.9 | 0.391 |
| 5 | 4.9 | 5.2 | 5.1 | 6.1 | 6.7 | 6.1 | 6.7 | 6.7 | 5.8 | 6.0 | <0.001 |
| 6 | 6.9 | 6.0 | 7.3 | 6.6 | 8.4 | 8.6 | 7.7 | 8.0 | 8.5 | 7.6 | 0.001 |
| P value | <0.001 | <0.001 | <0.001 | <0.001 | <0.001 | <0.001 | <0.001 | <0.001 | <0.001 | <0.001 |  |
| Sex |  |  |  |  |  |  |  |  |  |  |  |
| Boys | 5.2 | 5.3 | 5.5 | 5.6 | 6.5 | 6.2 | 6.0 | 6.1 | 5.9 | 5.8 | 0.001 |
| Girls | 3.1 | 3.1 | 3.2 | 3.2 | 3.8 | 3.7 | 4.0 | 3.7 | 3.7 | 3.5 | 0.001 |
| P value | <0.001 | <0.001 | <0.001 | <0.001 | <0.001 | <0.001 | <0.001 | <0.001 | <0.001 | <0.001 |  |
| Region |  |  |  |  |  |  |  |  |  |  |  |
| Central urban | 4.0 | 4.1 | 4.1 | 3.9 | 4.8 | 4.5 | 4.7 | 4.8 | 4.1 | 4.4 | 0.046 |
| New urban | 4.8 | 4.5 | 5.3 | 5.3 | 6.1 | 5.7 | 5.5 | 5.4 | 5.7 | 5.4 | 0.015 |
| Suburban | 3.9 | 4.5 | 4.1 | 4.4 | 4.8 | 5.1 | 5.1 | 5.1 | 4.8 | 4.7 | 0.005 |
| Rural area | 4.5 | 3.7 | 3.6 | 4.5 | 5.2 | 4.9 | 5.0 | 5.4 | 5.5 | 4.8 | 0.007 |
| P Value | 0.157 | 0.529 | 0.024 | 0.016 | 0.019 | 0.034 | 0.296 | 0.380 | 0.001 | <0.001 |  |
